# Strategies to prevent catheter-associated urinary tract infections in acute-care hospitals: 2022 Update

**DOI:** 10.1017/ice.2023.137

**Published:** 2023-08-25

**Authors:** Payal K. Patel, Sonali D. Advani, Aaron D. Kofman, Evelyn Lo, Lisa L. Maragakis, David A. Pegues, Ann Marie Pettis, Sanjay Saint, Barbara Trautner, Deborah S. Yokoe, Jennifer Meddings

**Affiliations:** 1 Division of Infectious Diseases, Intermountain Health, Salt Lake City, Utah, United States; 2 Division of Infectious Diseases, Duke University School of Medicine, Durham, North Carolina, United States; 3 Division of Healthcare Quality Promotion, Centers for Disease Control and Prevention, Atlanta, Georgia, United States; 4 St. Boniface General Hospital and University of Manitoba, Winnipeg, Manitoba, Canada; 5 Johns Hopkins University School of Medicine, The Johns Hopkins Hospital, Baltimore, Maryland, United States; 6 Division of Infectious Diseases, Perelman School of Medicine at the University of Pennsylvania, Hospital of the University of Pennsylvania, Philadelphia, Pennsylvania, United States; 7 University of Rochester Medicine, Rochester, New York, United States; 8 Department of Internal Medicine, University of Michigan Medical School, Ann Arbor, Michigan, United States; 9 Department of Medicine and the Center for Clinical Management Research, Veterans’ Affairs Ann Arbor Healthcare System, Ann Arbor, Michigan, United States; 10 Department of Internal Medicine, Baylor College of Medicine, Houston, Texas, United States; 11 Section of Health Services Research and the Center for Innovations in Quality, Effectiveness, and Safety, Michael E. DeBakey Veterans’ Affairs Medical Center, Houston, Texas, United States; 12 University of California San Francisco School of Medicine, UCSF Health-UCSF Medical Center, San Francisco, California, United States; 13 Department of Pediatrics, University of Michigan Medical School, Ann Arbor, Michigan, United States

## Abstract

The intent of this document is to highlight practical recommendations in a concise format designed to assist physicians, nurses, and infection preventionists at acute-care hospitals in implementing and prioritizing their catheter-associated urinary tract infection (CAUTI) prevention efforts. This document updates the *Strategies to Prevent Catheter-Associated Urinary Tract Infections in Acute-Care Hospitals* published in 2014. It is the product of a collaborative effort led by SHEA, the Infectious Diseases Society of America (IDSA), the Association for Professionals in Infection Control and Epidemiology (APIC), the American Hospital Association (AHA), and The Joint Commission.

## Summary of major changes

This section lists major changes provided in the *Strategies to Prevent Catheter-Associated Urinary Tract Infections in Acute-Care Hospitals: 2022 Update*,^1^ including recommendations that have been added, removed, or altered. Recommendations in this document are categorized as “essential practices” that are foundational to all HAI programs in acute-care hospitals (in 2014, these were termed “basic practices”) or “additional approaches” to be considered for use in locations and/or populations within hospitals during outbreaks in addition to full implementation of essential practices (in 2014 these were termed “special approaches”). See Table 1 for a complete summary of the recommendations contained in this document.

### Essential practices

- Updates to the implementation of evidence-based appropriateness criteria for indwelling urethral catheter use
- Discussion of strategies for urine-culture stewardship and their impact on CAUTI rates
- Updated performance measures to highlight the effects on catheter harm in addition to CAUTI
- Discussion of limitations of external urinary catheters

### Additional approaches

- Considerations for injury from urinary catheter use (ie, catheter harm) as well as non–catheter-associated urinary tract infections (eg, UTIs associated with use of alternative urinary collection devices such as external urinary catheters).
- An updated visual framework for “Disrupting the Life Cycle of Indwelling Urethral Catheter” has been provided to help identify where patient safety interventions can help reduce catheter-associated infection and trauma.

### Unresolved issues

- Standard of care for routine replacement of urinary catheters in place >30 days for the purpose of infection prevention.
- Best practices for optimizing and tailoring implementation of CAUTI prevention and urine-culture stewardship from the adult acute-care setting to the pediatric acute-care setting.

## Intended use

This document was developed following the process outlined in the *Handbook for SHEA-Sponsored Guidelines and Expert Guidance Documents*.^2^ No guideline or expert guidance document can anticipate all clinical situations, and this document is not meant to be a substitute for individual clinical judgement by qualified professionals. This document focuses on prevention of catheter-associated urinary tract infections (CAUTIs) in acute-care hospitals. The strategies highlighted may or may not be applicable for other healthcare settings, such as ambulatory settings or long-term or postacute-care facilities. Furthermore, there may be differences in healthcare environments within the hospital (eg, acute-care wards vs intensive care units vs perioperative spaces, etc) that may affect the feasibility of specific recommendations, which should be considered by stakeholders implementing these strategies.

This document is based on a synthesis of evidence, theoretical rationale, current practices, practical considerations, writing group consensus, and consideration of potential harm, where applicable. A summary of the recommendations is provided in Table 1.

## Methods

SHEA recruited 3 subject-matter experts in CAUTI prevention to lead the panel of members representing the Compendium partnering organizations: SHEA, IDSA, APIC, AHA, and The Joint Commission, as well as the Centers for Disease Control and Prevention (CDC).

SHEA utilized a consultant medical librarian who worked with the panel to develop a comprehensive search strategy for PubMed and Embase (January 2012–July 2019; updated to August 2021). Article abstracts were reviewed by panel members in a double-blind fashion using Covidence abstract management software (Covidence, Melbourne, Australia). The articles were subsequently reviewed as full text. The Compendium Lead Authors group voted to update the literature findings, and the librarian reran the search to update it to August 2021. Panel members reviewed the abstracts of these articles via Covidence and incorporated relevant references.

Recommendations resulting from this literature review process were classified based on the quality of evidence and the balance between desirable and potential undesirable effects of various interventions (Table 2). Panel members met via video conference to discuss literature findings, recommendations, quality of evidence for these recommendations, and classification as essential practices, additional approaches, or unresolved issues. Panel members reviewed and approved the document and its recommendations.

The Compendium Expert Panel, made up of members with broad healthcare epidemiology and infection prevention expertise, reviewed the draft manuscript after consensus had been reached by writing panel members.

Following review and approval by the expert panel, the 5 Compendium Partners, stakeholder organizations, and CDC reviewed the document. Prior to dissemination, the guidance document was reviewed and approved by the SHEA Guidelines Committee, the IDSA Standards and Practice Guidelines Committee, AHA, and The Joint Commission, and the Boards of SHEA, IDSA, and APIC. All members complied with SHEA and IDSA policies on conflict-of-interest disclosure.

## Section 1: Rationale and statements of concern

### Burden of outcomes associated with CAUTI

1. Urinary tract infections (UTIs) are one of the most common healthcare-associated infections. In 2003, 70%–80% of UTIs were attributable to the presence of an indwelling urethral catheter. In a 2019 analysis, over 5 years, CAUTIs decreased in proportion to non–device-associated UTIs but still made up an average of 44% of these infections per year among the hospitalized patients included in the study.^3,4^ The burden of CAUTI in pediatric patients is not well defined; however, bundles adapted from work in adults have been applied to the pediatric population with favorable results.^5^
2. Urinary catheters remain one of the most common medical devices experienced by adults in emergency departments and hospitals worldwide. Often, these devices are placed and maintained in use without an appropriate clinical indication to justify the risk compared to the benefit.^6–10^ Also, 12%–16% of adult hospital inpatients will have an indwelling urethral catheter at some point during admission.^11^ Of patients who have a urinary catheter placed in the hospital, up to half are placed in patients who may not have an appropriate indication for a urinary catheter.^12^
3. The daily risk of development of bacteriuria varies from 3% to 7% when an indwelling urethral catheter remains in situ.^13^
4. The high frequency of catheter use in hospitalized patients means that the cumulative burden of CAUTI is substantial.^3,14–16^
5. Infection is only one of several adverse outcomes of urinary catheter use. Noninfectious complications include nonbacterial urethral inflammation, urethral strictures, mechanical trauma, and mobility impairment, and these are described in this document as well. The CAUTI rates reported in 2020 for facilities reporting to the National Healthcare Safety Network (NHSN) were 0.754 per 1,000 catheter days for adult inpatient units. At one VA hospital, 0.3% of catheter days involved symptomatic UTI.^17^
6. Previous research has shown that CAUTI rates in intensive care units (ICUs) that reported to the NHSN ranged from 1.2 to 4.5 per 1,000 urinary catheter days in adult ICUs and from 1.4 to 3.1 per 1,000 urinary catheter days in pediatric ICUs.^18^ An 8% reduction was observed nationally in CAUTI incidence reported between 2018 and 2019, with the largest decrease noted in ICUs.^19,20^ The coronavirus disease 2019 (COVID-19) pandemic has affected HAI rates around the world, and data on changes in CAUTI rates were mixed during the pandemic.^21,22^
7. Bacteremia secondary to CAUTI is infrequent, as demonstrated in a review of 444 episodes of catheter-associated bacteriuria in 308 patients with CAUTI, in which only 3 patients (0.7%) developed bacteremia from a urinary source.^23^
8. CAUTI has been associated with increased mortality and length of stay, but the association with mortality may be a consequence of confounding by unmeasured clinical variables.^24^ The attributable costs of a CAUTI range from US$603 to US$1,189 for inpatients and up to US$1,764 for patients in ICUs.^25,26^

### Organizational outcomes associated with CAUTI

Inappropriate use of urine cultures can increase the rates of CAUTI according to the NHSN.^27,28^ In this document, “NHSN CAUTI” indicates ongoing, thorough review of medical and/or nursing notes in the electronic health record to observe the numbers of urinary catheters in place, urine cultures ordered, and prescribed antimicrobials. Inappropriate treatment of catheter-associated asymptomatic bacteriuria can promote antimicrobial resistance and *Clostridioides difficile* infection in acute-care facilities. Diagnostic stewardship strategies could be effective in the treatment and prevention of NHSN CAUTI.^27,29,30^

When interventions are implemented to prevent CAUTI, improved outcomes expected are reduced CAUTI, reduced indwelling urethral catheter use, reduced collection and antibiotic use for positive urine cultures, reduced antibiotic-associated complications, and reduced costs associated with these outcomes.

### Risk factors for CAUTI

1. The duration of catheterization is the most important risk factor for developing infection.^31–33^ Accordingly, reducing unnecessary catheter placement and minimizing the duration of catheterization are the primary strategies for CAUTI prevention.
2. Additional risk factors include female sex, older age, and not maintaining a closed drainage system.^34,35^ In pediatrics, specific clinical scenarios are often thought to require an open drainage system, including those with recent complex surgical repair or reconstruction of congenital abnormalities of the urogenital system. Available data are insufficient to determine whether specific patients would benefit more from an open drainage system despite an increased risk of CAUTI.^36,37^
3. Risk factors for developing healthcare-associated urinary-tract–related bloodstream infection include neutropenia, renal disease, and male sex.^38–40^

### Reservoirs of transmission

1. The drainage bag of the bacteriuric patient is a reservoir for organisms that may be transmitted through the hands of healthcare personnel (HCP).^41^
2. The drainage bag can also become contaminated by contact with hands due to inadequate hand hygiene, contact with the patient’s skin or hands, or contact with the floor or vessel used to empty the bag.
3. Outbreaks of infections associated with resistant gram-negative organisms attributable to bacteriuria in catheterized patients have been reported.^42–44^

## Section 2: Background on definitions of CAUTI

The clinical diagnosis of CAUTI is often a diagnosis of exclusion,^35^ making it difficult to have a standardized definition. At present, all of the available definitions have substantial limitations.^45^ We discuss the criteria, advantages, and limitations of different definitions used for CAUTI in Table 4.

The optimal definition for CAUTI used for surveillance and quality improvement is one that only captures true instances of symptomatic infection that would benefit from antimicrobial treatment. The NHSN CAUTI definition has been adopted nationally, but other definitions are also used for clinical care and administrative purposes.^46^ The NHSN CAUTI definition has been updated in 2015 with exclusion of yeast as a pathogen and increase in the urine-culture bacterial threshold to ≥10^5^ colony-forming units per milliliter (CFU/mL) to align with a clinical definition for symptomatic CAUTI.^47,48^

## Section 3: Background on prevention of CAUTI

### Summary of existing guidelines and recommendations

(See Supplementary Content, Appendix 1 online)

1. The CDC published guidelines for prevention of CAUTI in 1981, and these were updated in 2009.^34^ These guidelines provide recommendations for catheter use, catheter insertion, catheter care, and implementation of programs to prevent CAUTI.
2. The CDC also developed the Targeted Assessment for Prevention (TAP) Strategy as a framework for quality improvement that uses data for action to prevent HAIs,^49^ including CAUTIs. The following 3 components of the TAP Strategy focus on CAUTI:
  - Running TAP reports in the NHSN to target healthcare facilities and specific units with excess CAUTIs.
  - Applying TAP Facility Assessment Tools to identify gaps in infection prevention in the targeted locations.
  - Accessing infection prevention resources within the TAP CAUTI Implementation Guide^49^ to address identified gaps in CAUTI prevention.
3. The IDSA together with other professional societies published international guidelines for the management of CAUTI in 2010.^50^
4. The Department of Health in Great Britain published guidelines for preventing infections associated with the insertion and maintenance of short-term indwelling urethral catheters in acute care in 2001,^51^ updated in 2014.^52^
5. Pragmatic tools for reducing inappropriate use of indwelling urethral catheters and antibiotics have also been published by organizations of hospitalists,^53^ nurses, and other funders of interventions to improve safety are also readily accessible for use, including resources targeting acute-care, long-term care, and ambulatory settings.

### Conceptual models and frameworks for prioritizing interventions to prevent CAUTI

**Figure 1.**
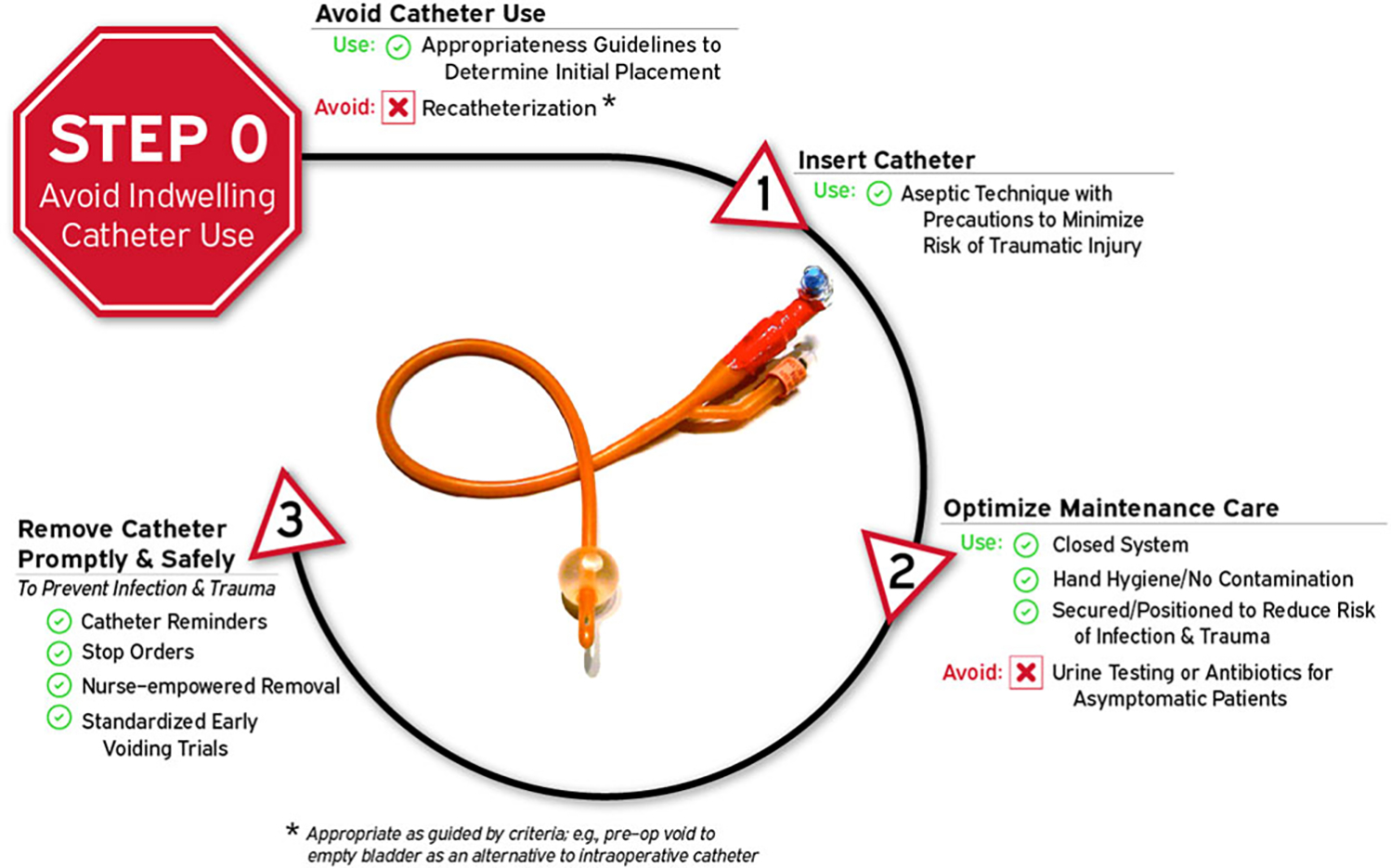
Disrupting the life cycle of the indwelling urethral catheter to reduce catheter-associated infection and trauma.

Given the number of intervention opportunities for reducing urinary-catheter–associated complications, a conceptual model known as “Disrupting the Life Cycle of the Urinary Catheter” may help to assess the comprehensiveness of a hospital’s or unit’s strategies for preventing catheter-associated complications, including CAUTI.^54^ This conceptual model has been used in recent large-scale collaboratives funded by the CDC and the Agency for Healthcare Research and Quality (AHRQ) in teaching materials to summarize critical types of interventions to consider in a comprehensive CAUTI prevention program.^55–57^ As illustrated in Figure 1 and adapted for this current document, the most important step (Life Cycle Step 0, labeled as Step 0 because it occurs before a urinary catheter’s “life” (or existence) as a medical device placed in the patient) to prevent both infectious and noninfectious catheter complications is avoiding placement of the indwelling catheter whenever possible. Life Cycle Step 0 includes employing non-catheter urinary management strategies such as prompted toileting, urinals, bedside commodes, and incontinence garments, and/or non–indwelling-catheter strategies such as intermittent straight catheterization (ISC) or consideration of external urinary catheters (EUCs).^58,59^ Literature for EUCs related to cost-effectiveness, risks of EUC–associated skin injury or UTIs compared to other strategies is still evolving.^58–62^ Refer to Table 5 for summary of recent literature on CAUTI prevention initiatives.

## Section 4: Recommended strategies for CAUTI prevention

Recommendations are categorized either as essential practices that should be adopted by all acute-care hospitals or as additional approaches that can be considered for use in locations and/or populations within hospitals when CAUTIs are not controlled using essential practices. Essential practices include recommendations in which the potential to impact CAUTI risk clearly outweighs the potential for undesirable effects. Additional approaches include recommendations in which the intervention is likely to reduce CAUTI risk but there is concern about the risks for undesirable outcomes, the quality of evidence is low, or evidence supports the impact of the intervention in select settings (eg, during outbreaks) or for select patient populations. Hospitals can prioritize their efforts by initially focusing on implementing essential practices. If CAUTI surveillance or other risk assessments suggest that ongoing opportunities for improvement exist, hospitals should then consider adopting some or all of the additional approaches. These interventions can be implemented in specific locations or patient populations or can be implemented hospital-wide, depending on outcome data, risk assessment, and/or local requirements. Each infection prevention recommendation is evaluated for quality of evidence (Table 2). Recommendations for preventing and monitoring CAUTI^34,35,51,52^ are summarized in the following section and Table 1.

### Essential practices for preventing CAUTI: Recommended for all acute-care hospitals

1. Perform a CAUTI risk assessment and implement an organization-wide program to identify and remove catheters that are no longer necessary using 1 or more methods documented to be effective.^34,35,51,52^ (Quality of evidence: MODERATE)
  - Develop and implement institutional policy requiring periodic, usually daily, review of the necessity of continued catheterization.
  - Consider utilizing electronic or other types of reminders (see Supplementary Content, Appendices 2 and 3 online) of the presence of a catheter and required criteria for continued use.^63^ Examples include the following:
    - Automatic stop orders requiring review of current indications and renewal of order for continuation of the indwelling catheter.
    - Standardized reminders of persistent catheters together with current catheter indications (see Supplementary Content, Appendices 2 and 3 online) targeting either physicians or nurses.
  - Nursing and physician staff conduct daily reviews during rounds of all patients with urinary catheters to ascertain necessity of continuing catheter use.^64^
2. Provide appropriate infrastructure for preventing CAUTI.^56^ (Quality of evidence: LOW)
  - Ensure that the supplies for following best practices for managing urinary issues are readily available to staff in each unit, including bladder scanners, non–catheter-incontinence management supplies (urinals, garments, bed pads, skin products), male and female external urinary catheters, straight urinary catheters, and indwelling catheters including the option of catheters with coude tips.
  - Ensure that non-catheter urinary management supplies are as easy to obtain for bedside use as indwelling urinary catheters.
  - Ensure the physical capability for urinary catheters with tubes attached to patients (eg, indwelling urinary catheters and some external urinary catheters [EUCs]) to be positioned on beds, wheelchairs, at an appropriate height and without kinking for patients in their rooms and during transport.
3. Provide and implement evidence-based protocols to address multiple steps of the urinary catheter life cycle (Fig. 1): catheter appropriateness (step 0), insertion technique (step 1), maintenance care (step 2), and prompt removal (step 3) when no longer appropriate. (Quality of evidence: LOW)
  - Adapt and implement evidence-based criteria for acceptable indications for indwelling urethral catheter use, which may be embedded as standardized clinical-decision support tools within electronic medical record (EMR) ordering systems. Expert-consensus–derived indications for indwelling catheter use have been developed, although research that assesses the appropriateness of these uses is limited.^34,65^ The limited examples of appropriate indications for indwelling urethral catheter include the following:
    - Perioperative use for selected surgical procedures^66,67^ such as urologic surgery or surgery on contiguous structures of the genitourinary tract, prolonged surgery, large-volume infusions or diuretics during surgery, or intraoperative monitoring of urine output is needed. Notably, if a catheter is placed intraoperatively simply due to the duration of surgery (eg, >3 hours) or for decompression to address a specific surgical approach, then such catheters should be removed at the end of the surgical case.
    - Hourly assessment of urine output in ICU patients when used clinically to modify therapies frequently such as volume resuscitation, diuresis, and vasopressors. ICU care alone is not an appropriate justification for indwelling catheter placement; a specific clinical indication is still needed.
    - Management of acute urinary retention^65,68^ (eg, new retention of urine with postvoid residual bladder volume >500 cm^3^ by bladder scanner if no symptoms, or >300 cm^3^ if having symptoms such as bladder pain or fullness, persistent urge to void, new incontinence or leaking, or only able to have frequent small voids)
    - Assistance in healing of open pressure ulcers or skin grafts for selected patients with urinary incontinence when alternative supplies for protective wound or managing incontinence (eg, external urinary catheters, wound dressings) are not feasible.
    - Facilities may allow exceptions as part of a palliative and/or comfort care regimen, if use of the catheter addresses a specific goal of the patient, such as reducing the need for frequent bed or garment changes or preventing pain that cannot be well managed.
4. Ensure that only trained HCP insert urinary catheters and that competency is assessed regularly.^65^ (Quality of evidence: LOW)
  - Require supervision by experienced HCP when trainees insert and remove catheters to reduce the risk of infectious and traumatic complications related to urinary catheter placement.^69–71^ Given much higher rates of CAUTI when catheters are placed by trainees such as medical students,^71,72^ educational programs may need to reassess at which point in medical training and which trainees specifically are most appropriate for being involved in urinary catheter insertion in patients compared to simulation models only.
5. Ensure that supplies necessary for aseptic technique for catheter insertion are available and conveniently located. (Quality of evidence: LOW)
6. Implement a system for documenting the following in the patient record: physician order for catheter placement, indications for catheter insertion, date and time of catheter insertion, name of individual who inserted catheter, nursing documentation of placement, daily presence of a catheter and maintenance care tasks, and date and time of catheter removal. Record criteria for removal and justification for continued use. (Quality of evidence: LOW)
  - Record in a standard format for data collection and quality improvement purposes and keep accessible documentation of catheter placement (including indication) and removal.
  - If available, utilize electronic documentation that is searchable.
  - Consider nurse-driven urinary catheter removal protocols for first trial of void without an indwelling catheter when the indication for placement has resolved (see Essential Practices, 3). These protocols can be implemented as part of the routine urinary catheter placement order or as an expected reminder (or “standing order”) from the nurse to the physician in rounds. These protocols should list some exceptions or “opt outs,” such as for postoperative urology surgery patients or patients for whom a catheter required urology consult for placement that should not be removed without physician order.
7. Ensure that sufficiently trained HCP and technology resources are available to support surveillance for catheter use and outcomes.^73^ (Quality of evidence: LOW)
8. Perform surveillance for CAUTI if indicated based on facility risk assessment or regulatory requirements as described in Section 5.^73^ (Quality of evidence: LOW)
9. Standardize urine culturing by adapting an institutional protocol for appropriate indications for urine cultures in patients with and without an indwelling catheter.^27,53,74–76^ Consider incorporating these indications into the EMR, and review indications for ordering urine cultures in CAUTI risk assessment.^77^ (Quality of evidence: LOW)

### Education and training

1. Educate HCP involved in the insertion, care, and maintenance of urinary catheters about CAUTI prevention, including alternatives to indwelling catheters, and procedures for catheter insertion, management, and removal.^78^ (Quality of evidence: LOW)
2. Assess healthcare professional competency in catheter use, catheter care, and maintenance.^79–81^ (Quality of evidence: LOW)
3. Educate HCP about the importance of urine culture stewardship and provide indications for urine cultures. (Quality of evidence: LOW)
  - Consider requiring clinicians to identify an appropriate indication for urine culturing when placing an order for a urine culture. The indication should be supported by the literature^29,82–86^ and should be appropriate for the specific patient population. Include guideline-based reminders^87^ for the specific circumstances. Below is a simple example of appropriate and inappropriate reasons to culture urine are referenced online on the CDC website,^82,83^ though several other lists are available in the literature specific to particular clinical settings (eg, ICU, emergency department, nursing home, and catheterized versus non-catheterized patients):
    - Appropriate uses of urine culture include the following:
      - Presence of symptoms suggestive of a urinary tract infection (UTI):
        - Flank pain or costovertebral angle tenderness
        - Acute hematuria
        - New pelvic discomfort
      - New onset or worsening sepsis without evidence of another source on history, physical examination, or laboratory testing
      - Fever or altered mental status without evidence of another source on history, physical examination, or laboratory testing
      - In spinal-cord-injury patients and other highly complex patients (eg, patients with >40% total body burn, recipients of kidney transplants with graft failure) symptoms may include increased spasticity, autonomic dysreflexia, and/or sense of unease.
    - Inappropriate uses of urine cultures include the following:
      - Odorous, cloudy, or discolored urine in the absence of other localizing signs and symptoms
      - Reflex urine cultures based on urinalysis results, such as pyuria, in the absence of other indications (absence of pyuria suggests diagnosis other than CAUTI)
      - Urine culture to document response to therapy unless symptoms fail to resolve.
4. Provide training on appropriate collection of urine. Specimens should be collected and should arrive at the microbiology laboratory as soon as possible, preferably within an hour. If delay in transport to the laboratory is expected, samples should be refrigerated (no more than 24 hours) or collected in preservative urine transport tubes. (Quality of evidence: LOW)
5. Train clinicians to consider other methods for bladder management (eg, intermittent catheterization, or external male or female collection devices) when appropriate before placing an indwelling urethral catheter. (Quality of evidence: LOW)
6. Share data in a timely fashion and report to appropriate stakeholders. (Quality of evidence: LOW)

### Insertion of indwelling catheters

1. Insert urinary catheters only when necessary for patient care and leave in place only as long as indications remain. (Quality of evidence: MODERATE)
2. Consider other methods for bladder management such as intermittent catheterization, or external male or female collection devices, when appropriate. (Quality of evidence: LOW)
3. Use appropriate technique for catheter insertion. (Quality of evidence: MODERATE)
4. Consider working in pairs to help perform patient positioning and monitor for potential contamination during placement.^88–90^ (Quality of evidence: LOW)
5. Practice hand hygiene (based on CDC or World Health Organization guidelines) immediately before insertion of the catheter and before and after any manipulation of the catheter site or apparatus. (Quality of evidence: LOW)
6. Insert catheters following aseptic technique and using sterile equipment. (Quality of evidence: LOW)
7. Use sterile gloves, drape, and sponges, a sterile antiseptic solution for cleaning the urethral meatus, and a sterile single-use packet of lubricant jelly for insertion. (Quality of evidence: LOW)
8. Use a catheter with the smallest feasible diameter consistent with proper drainage to minimize urethral trauma but consider other catheter types and sizes when warranted for patients with anticipated difficult catheterization to reduce the likelihood that a patient will experience multiple, sometimes traumatic, catheterization attempts. (Quality of evidence: LOW)

### Management of indwelling catheters

1. Properly secure indwelling catheters after insertion to prevent movement and urethral traction.^91,92^ (Quality of evidence: LOW)
2. Maintain a sterile, continuously closed drainage system.^92,93^ (Quality of evidence: LOW)
3. Replace the catheter and the collection system using aseptic technique when breaks in aseptic technique, disconnection, or leakage occur. (Quality of evidence: LOW)
4. For examination of fresh urine, collect a small sample by aspirating urine from the needleless sampling port with a sterile syringe or cannula adaptor after cleansing the port with disinfectant. (Quality of evidence: LOW)
5. Facilitate timely transport of urine samples to laboratory. If timely transport is not feasible, consider refrigerating urine samples or using sample collection cups with preservatives. Obtain larger volumes of urine for special analyses (eg, 24-hour urine) aseptically from the drainage bag. (Quality of evidence: LOW)
6. Maintain unobstructed urine flow. (Quality of evidence: LOW)
  - Remind bedside caregivers, patients, and transport personnel to always keep the collecting bag below the level of the bladder.
  - Do not place the bag on floor.
  - Keep the catheter and collecting tube free from kinking, which can impair urinary flow and increase stasis within the bladder, increasing infection risk.
  - Empty the collecting bag regularly using a separate collecting container for each patient. Avoid touching the draining spigot to the collecting container.
7. Employ routine hygiene. Cleaning the meatal area with antiseptic solutions is an unresolved issue, though emerging literature supports chlorhexidine use prior to catheter insertion.^94–97^ Alcohol-based products should be avoided given concerns about the alcohol causing drying of the mucosal tissues (Photos of these steps are available).^92,93^ (Quality of evidence: LOW)

### Additional approaches for preventing CAUTI

These additional approaches are recommended for use in locations and/or populations within the hospital with unacceptably high CAUTI rates or standardized infection ratios (SIRs) despite implementation of the essential CAUTI prevention strategies listed previously.

**Figure 2.**
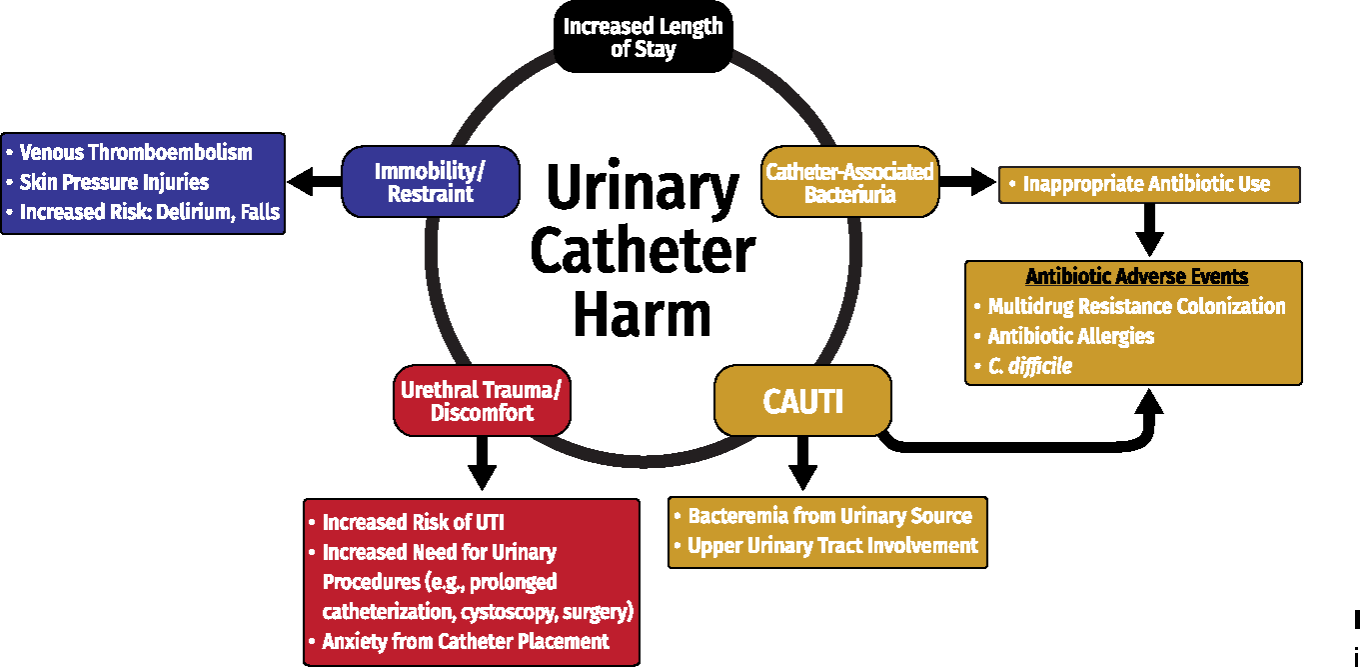
Infectious and noninfectious complications of an indwelling urethral catheter.

1. Develop a protocol for standardizing diagnosis and management of postoperative urinary retention, including nurse-directed use of intermittent catheterization and use of bladder scanners^98,99^ when appropriate as alternatives to indwelling urethral catheterization. (Quality of evidence: MODERATE)
  - If bladder scanners are used, clearly state indications, train nursing staff in their use, and disinfect between patients according to manufacturer’s instructions.
2. Establish a system for analyzing and reporting data on catheter use and adverse events from catheter use. (Quality of evidence: LOW)
  - Use cumulative attributable difference to identify high-risk units or hospitals (as described in Section 5).
  - Measure process and outcomes measures (eg, standardized utilization ratio and standardized infection ratio) as described in Section 5.
  - Define and monitor catheter harm (see Fig. 2) in addition to CAUTI, including catheter obstruction, unintended removal, catheter trauma, or reinsertion within 24 hours of removal.^100^
    - National focus has been on CAUTI prevention, but current metrics do not adequately capture overall catheter harm.^101^ Catheter harm includes infectious complications in addition to CAUTI (eg, secondary bacteremia, asymptomatic bacteriuria consequences) and noninfectious catheter complications (Fig. 2).
    - Patients with an indwelling urethral catheter are 5 times more likely to experience noninfectious complications (eg, urethral injury, pain, or inadvertent catheter removal) than infectious complications.^100^
    - Current metrics used to monitor progress in the prevention of CAUTIs may underestimate both infectious and noninfectious catheter harm.
3. Establish a system for defining, analyzing, and reporting data on non–catheter-associated UTIs, particularly UTIs associated with devices used as alternatives to indwelling urethral catheters. (Quality of evidence: LOW)
  - Non–catheter-associated UTIs are defined as UTIs that occur in hospitalized patients without an indwelling urethral catheter. These include but are not limited to patients who have had no urinary device at all, as well as those with external urinary catheters (EUCs), urinary stents, or urostomies, or who undergo intermittent catheterization, and thus are not captured by the NHSN CAUTI definition.
  - As the incidence of CAUTI continues to decline, the proportion of non–catheter-associated UTIs is increasing in some hospitals.^4^ However, the national incidence of non–catheter-associated UTIs is not known because surveillance and reporting of these UTIs are not required by US Federal agencies.
  - Non–catheter-associated UTIs are a common indication for antibiotics in hospitalized patients, and this metric could provide important information as healthcare facilities consider the risks and benefits of newer alternatives to urinary catheters with currently limited published data on adverse events (eg, EUCs for women) to help inform when the benefit outweighs the potential risk for specific patient populations.

### Approaches that should not be considered a routine part of CAUTI prevention

1. Routine use of antimicrobial- or antiseptic-impregnated catheters. (Quality of evidence: HIGH)
2. Breaking a closed system. (Quality of evidence: LOW)
3. Screening for asymptomatic bacteriuria in catheterized patients, except in the few patient populations for which this is anticipated to have more benefit than harm, as detailed in the 2019 IDSA Guideline for Management of Asymptomatic Bacteriuria^102^ and the 2019 US Preventative Services Task Force Recommendation on Asymptomatic Bacteriuria in Adults^102^ (eg, pregnant women, patients undergoing endoscopic urologic procedures associated with mucosal trauma). (Quality of evidence: HIGH)
  - Treatment of asymptomatic bacteriuria is not an effective strategy to prevent CAUTI in other patient groups, as it increases the risk of antibiotic-associated complications more than any potential benefit for the prevention of symptomatic CAUTI. The conditions that predispose the patient to bladder colonization (anatomic, immunologic) are not resolved by antibiotics, so bacteriuria recurs.
4. Catheter irrigation as a strategy to prevent infection. (Quality of evidence: MODERATE)
  - Do not perform continuous irrigation of the bladder with antimicrobials as a routine infection prevention measure.
  - If continuous irrigation is being used to prevent obstruction, maintain a closed system.
5. Routine use of systemic antimicrobials as prophylaxis. (Quality of evidence: LOW)
6. Routine changing of catheters to avoid infection. (Quality of evidence: LOW)
  - In the case of a patient with a long-term catheter in place (ie, >7 days), catheter replacement can be considered at the time of specimen collection for urine testing to obtain a fresh sample.^103,104^
7. Alcohol-based products on the genital mucosa. (Quality of evidence: LOW)

### Unresolved issues and future areas of study

1. Use of antiseptic solution versus sterile saline for meatal and perineal cleaning prior to catheter insertion.^94–96,105^
2. Use of urinary antiseptics (eg, methenamine) to prevent UTI.
3. Spatial separation of patients with urinary catheters in place to prevent transmission of pathogens that could colonize urinary drainage systems.
4. Standard of care for routine replacement of urinary catheters in place >30 days for infection prevention.^106^
5. Best practices for optimizing and tailoring implementation of CAUTI prevention and urine-culture stewardship from the adult acute-care setting to the pediatric acute-care setting.

## Section 5: Performance measures

Different performance measures have been used for internal and external reporting of CAUTIs and catheter utilization. Hospitals may use a combination of metrics for public reporting and to assess the impact of quality improvement initiatives. Here, we discuss outcome and process measures for both internal and external reporting.

### Internal reporting

These performance measures are intended to support internal hospital quality improvement efforts and do not necessarily address external reporting requirements. The process and outcome measures suggested here are derived from published guidelines, other relevant literature, and the opinions are those of the authors. These process and outcome measures can be shared with senior hospital leadership, nursing leadership, and clinicians who care for patients at risk for CAUTI.^107,108^

### Process measures

1. Percentage of inappropriate catheters based on insertion documentation
  - Conduct random audits of selected units and calculate utilization ratios:
    - Numerator: number of patients on the unit with a urinary catheter and an inappropriate indication for the catheter.
    - Denominator: number of patients on the unit with a urinary catheter in place.
    - Multiply by 100 so that the measure is expressed as a percentage.
2. Percent compliance with daily documentation of continued need for indwelling urethral catheter
  - Measure the percentage of patients with an indwelling catheter and documentation of daily assessment of need
    - Numerator: number of patients with an indwelling catheter who have documentation of daily assessment.
    - Denominator: number of patients with an indwelling catheter.
    - Multiply by 100 so that the measure is expressed as a percentage.
3. Point prevalence of indwelling urethral catheters for a specific unit
  - Conduct audits of all units and calculate the device utilization ratio^109^
    - Numerator: total number of urinary catheter days for all patients in a unit who have an indwelling urethral catheter.
    - Denominator: total number of patient days for all patients in a unit who are monitored.
    - Divide the numerator by the denominator to get point prevalence of presence of indwelling urethral catheters (for a specific unit).

### Outcome measures

1. CAUTI rates, stratified by risk factors (eg, ward, clinical service line)
  - Measure CAUTI rates over time to gauge the longitudinal impact of prevention strategies^34^
    - Numerator: number of CAUTIs in a unit that is monitored.
    - Denominators
      1. Total number of urinary catheter days for all patients on the unit who have an indwelling urethral catheter.
      2. Total number of patient days for all patients in a unit that is monitored.
    - Multiply by 1,000 so that the measure is expressed as cases per 1,000 catheter days, or 10,000 to express as cases per 10,000 patient days.^110^ Patient days may be better suited as the denominator in locations with significant reductions in catheter use or changes in risk profile (eg, removing low-risk catheters in ED or ICU leaves a population of high-risk catheters).^111^
2. Standardized infection ratio (SIR)
  - The SIR is a risk-adjusted summary measure that allows for a comparison to the national benchmark and can be used to track CAUTI incidence over time.
  - The ratio is calculated by dividing the observed number of CAUTIs by the predicted number of CAUTIs.
  - The predicted number of infections is an estimated number of CAUTIs based on infections reported to NHSN during a baseline period (currently 2015 for CAUTI, risk-adjusted for patient care location and facility characteristics).
3. Cumulative attributable difference (CAD)
  - The cumulative attributable difference (CAD) is used in the CDC Targeted Assessment for Prevention (TAP) Strategy to target prevention efforts to hospitals or units with the highest excess HAIs.^112^ The CAD is the number of excess infections that need to be prevented to reach a goal SIR (set by the end user) as shown below:
    - CAD = observed number of CAUTIs – prevention target (predicted×SIR_goal_)
    - CAD is a cost-effective strategy used for targeting and prioritizing units.

The SIR is useful for evaluating relative risk differences, while the CAD is useful for evaluating absolute risk differences.

Internal reporting can be strengthened in the future by developing and refining metrics that target rate of urine culturing as well as compliance with urine collection techniques in patients with and without indwelling catheters.

### External reporting

There are many challenges in providing useful information to consumers and other stakeholders in public reporting of HAIs.^113^ Recommendations for public reporting of HAIs have been provided by the Healthcare Infection Control Practices Advisory Committee (HICPAC),^114^ the Healthcare-Associated Infection Working Group of the Joint Public Policy Committee, and the National Quality Forum (NQF). In January 2012, most acute-care facilities began reporting CAUTIs from adult and pediatric ICUs to the NHSN to meet requirements of the Centers for Medicare and Medicaid Services (CMS) Inpatient Prospective Payment System FY2012 final rule.

The validity of the current CDC NHSN definition for CAUTI for comparing facility-to-facility outcomes has not been established, so caution is recommended when performing interfacility comparisons of CAUTI rates. Use of hospital claims data to compare healthcare-associated CAUTI rates also has not been validated.^115^ Choice of metrics may also be influenced by hospital size. For example, SIR may be more suitable for larger hospitals or hospitals with higher CAUTI events, while SUR or “days since last CAUTI” may be a more useful metric for smaller hospitals or hospitals with rare events.^107,108^

### State and local requirements

Hospitals in states that have mandatory reporting requirements must collect and report the data required by the state. For information on state and federal requirements, check with your state or local health department.

### External quality initiatives

Hospitals that participate in external quality initiatives must collect and report the data required by the initiative.

### Outcome measures

1. Standardized utilization ratio (SUR): The SUR is the primary summary measure used to compare device utilization at the national, state, or facility level.
  - This ratio is calculated by dividing the observed number of device days by the predicted number of device days.
  - The predicted number of device days is an estimated number of device days based on data reported to NHSN during a baseline period (currently 2015 for CAUTI, risk-adjusted for patient care location and facility characteristics).
2. Standardized infection ratio (SIR): The SIR is a summary measure used to track HAIs at a national, state, or facility level over time, and has been described above.
  - Consider measures that address device risk at the patient population level. A population SIR (pSIR) accounts for both SIR and SUR, reflecting both device care and device utilization.

Using different metrics in combination is a better strategy than using a single metric to target prevention efforts. For example, hospitals with low SIRs and high device utilization may represent low-risk catheter use, better maintenance and care of indwelling catheters, or strict urine-culturing practices. In these scenarios, focusing prevention efforts on decreasing device utilization should be considered to account for noninfectious catheter harm as well. Alternatively, hospitals that have high SIRs and low device utilization may represent a population with more high-risk catheter use (ie, catheters in high-risk patients), inadequate catheter care, or indiscriminate urine culturing practices. These hospitals may benefit from focusing on catheter maintenance and stewardship of culturing.^107,108^

## Section 6: Implementation strategies

Preventing CAUTI requires a focus on both technical and socioadaptive (or behavioral) components.^116^ Although the general concepts of implementation science are found in a companion article of the 2022 Compendium, we detail here some of the CAUTI-specific implementation work—and lessons learned—those hospitals should be aware of since the 2014 update.^117^ Interventions to assist with program implementation and evaluation that have been reported to be associated with improved outcomes are provided in this section. These interventions are often multifaceted and generally include elements of the “4E’s” model.^118^ This section is particularly aimed toward physicians, nurses, and infection preventionists that are in leadership positions, to review when designing or revising strategies to optimize implementation of CAUTI prevention strategies.

Over the past several years, regional and national CAUTI prevention initiatives have been implemented in acute-care hospitals. Several of these initiatives have been successful^119–121^ but not in all patient care locations or settings.^56,122^ In 2013, investigators in Michigan published the results of a comparison study in which they surveyed infection preventionists nationally (with an oversample of Michigan hospitals) and also evaluated SIRs comparing Michigan with non-Michigan hospitals.^119^ In this study, hospitals that were more likely to participate in collaboratives aimed at reducing HAIs, more likely to use bladder scanners, stop orders, reminders, or nurse-initiated removal of urinary catheters, corresponded to a 25% reduction of CAUTI rates compared with other hospitals.^119^

In a study of 7 Veterans’ Affairs (VA) hospitals, a multidisciplinary team demonstrated a significant reduction in CAUTI rates on medical-surgical wards (from 2.4 to 0.8 CAUTI per 1,000 catheter days; *P* = .001) but no significant change in ICU CAUTI rates.^120^ The intervention used a 2-tier system of interventions to determine each hospital’s specific needs relative to their CAUTI rates. Tier 1 describes data-gathering needs and a nursing template in the EMR to prompt staff to consider removing catheters at the earliest possible point. This tier alone can create sufficient visibility within the organization to reduce CAUTI rates to the desired level. Tier 2 is a more intensive tier of steps that hospitals can implement for stubborn rates that will not come down, including root-cause analysis. In a separate study by Saint et al, the team implemented a multifactorial intervention at >900 non-VA hospital units in the United States. This intervention utilized the Comprehensive Unit-Based Safety Program (CUSP). As with the Saint VA hospital study, this intervention led to a reduction in CAUTI rates in non-VA hospital medical-surgical wards (from 2.40 to 2.05 CAUTIs per 1,000 catheter days) but not in ICUs.^121^ These 2 CAUTI prevention efforts in VA and non-VA hospitals have thus far failed to significantly reduce rates in ICUs, likely related to several reasons including cultural differences between critical-care units and medical-surgical floors in the United States. Specifically, critical-care units often consider indwelling urethral catheters to be a standard of care for their patients,^123^ even when suitable alternatives could be used.^65,123^ The CUSP model was also used to focus entirely on ICUs with elevated baseline rates of CAUTI; this negative study revealed a need to develop a new model for helping such struggling hospitals. In the nursing home setting, as part of the AHRQ Safety Program for Long-Term Care, use of a combined technical and socioadaptive intervention reduced CAUTI rates by 54% and culture orders by 15%.^124^

**Figure 3.**
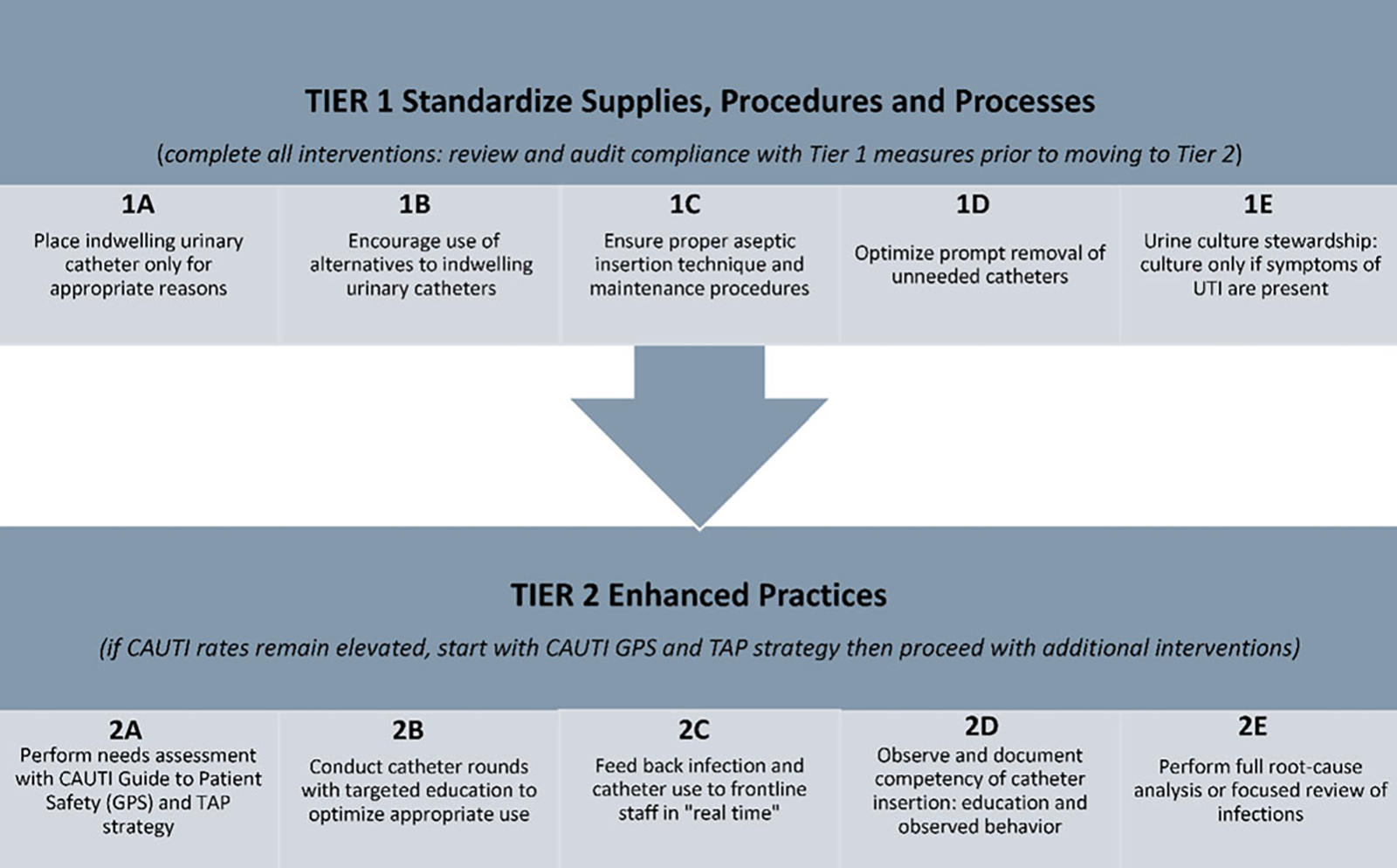
Tiered approach to reducing CAUTI.

The CDC recently funded a large-scale CAUTI intervention in medical-surgical units. This approach used 2 levels (or tiers) of interventions to approach and reduce persistently elevated CAUTI rates (Table 5 and Fig. 2). In short, the tier 1 interventions are applicable to all situations, regardless of CAUTI rates. However, if CAUTI rates remain elevated after implementing all 5 steps of tier 1 interventions, then the next tier is used. The process in tier 2 begins by using the *Guide to Patient Safety,*^125^ a validated tool developed to help HCP through the process of identifying barriers to reducing CAUTI rates. This tool uses a series of questions designed to help an organization identify areas for improvement coupled with evidence-based annotated responses to simple “yes or no” questions (Table 5 and Fig. 3; https://www.catheterout.org/cauti-gps.html).^126^

Additional technical processes are often needed for successful implementation. However, technical processes alone rarely are enough to effect change. Those processes must also be adapted to specific social aspects of a given organization. This distinction between technical and socioadaptive (or behavioral) strategies is critical to proper integration of new processes.

Through social adaptation of technical strategies, innovation can be diffused in an organization.^127^ Consider technical means to reduce infection (eg, ready availability of hand sanitizers or bladder scanners and automatic urinary catheter stop orders in the hospital’s electronic health record system) as well as social adaptations such as changing organizational culture or norms of clinical practice or engaging clinicians with CAUTI initiatives. During a recent survey, Greene et al^128^ collected data from infection preventionists across the United States. Part of the survey evaluated how feelings of psychological safety interact with patient safety goals. Psychological safety is defined as “the degree to which people view the environment as conducive to interpersonally risky behaviors like speaking up if they witness an error or asking for help if they have concerns about an order.”^128^ As an example of such a connection, they calculated the odds of regularly using technical initiatives for CAUTI reduction (eg, urinary catheter reminders) based on the degree of psychological safety the respondent felt in their organization. These researchers found high psychological safety (38% of respondents) to be associated with higher odds of using the technical initiatives (odds ratio, 2.37; *P* = .002).

Ensuring high psychological safety among HCP should also ideally be a foundational aspect of infection prevention practices. Similarly, survey results examining followership characteristics—personal attributes of those who follow a mentor, such as being energized by work, taking initiative, or independent thinking—demonstrated that the quality of follower has a direct influence on the uptake of recommended infection prevention practices.^129^ Frameworks like the Consolidated Framework for Implementation Research (CFIR) provide constructs across 5 domains: inner setting, outer setting, intervention, individuals, and process.^130^ These constructs provide a practical guide for assessing socioadaptive and technical aspects, diffusion of innovation, and program evaluation.

Investigators have also employed formal qualitative approaches to assess CAUTI implementation successes and challenges. For example, Krein et al^131^ examined the statewide impact of a “Bladder Bundle” initiative to reduce unnecessary use of urinary catheters. The team gathered qualitative data through semi-structured interviews and site visits, resulting in the identification of key barriers to successful CAUTI reduction: (1) lack of nurse and/or physician engagement in the program; (2) patient and/or family requests for catheters; and (3) emergency department (ED) customs and practices.^131^ Although these barriers represent important impediments, the investigators also identified strategies the participating hospitals used to address those barriers: (1) using urinary management (eg, planned toileting) in combination with other patient safety programs like fall prevention; (2) conversations with patients and/or families to clearly explain the risks associated with catheters; and (3) standardizing appropriateness criteria for staff working in the ED (to prevent placement of catheters for inappropriate reasons).^131^ In another qualitative study, Harrod et al^132^ examined the perception of risk of HCP and how that related to their use of infection prevention practices such as indwelling urethral catheter. These study findings indicated that patient risk is not the only consideration for these HCP when deciding whether to use a urinary catheter. The study identified several areas for potential improvement: (1) the need for HCP to deal with competing priorities and insufficient time; (2) identifying “gray areas” where the connection cannot be directly made between the use of a urinary catheter and a negative patient outcome; (3) process weakness in either or both existing organization policies and the new initiative being undertaken; and (4) HCP using “workarounds” to undermine the effectiveness of catheter use reduction programs.^132^ Investigators can thus tailor future interventions to address broader issues of risk to patient safety, realizing that objective risk from device use itself is not the only factor HCP consider when deciding to use a urinary catheter.

Implementation models like Capability–Opportunity–Motivation–Behavior (COM-B), Theoretical Domains Framework (TDF), and the Health Belief Model can be employed for better understanding of barriers and facilitators of CAUTI Prevention. Parker et al^133^ also examined the barriers to and facilitators of a successful CAUTI reduction program by conducting 8 focus groups with a total of 35 individuals. Their team identified several major themes based on the use of their “NO CAUTI” bundle.^134^ They identified 2 facilitators to this process: (1) early and sustained key stakeholder engagement and (2) effective advance planning that allows for adaptation during implementation. They also reported 2 barriers: (1) managing the change itself is a burden and (2) sustaining practice change is difficult and must be properly managed.^133^ An overview of practical approaches for problem solving regarding potential barriers to implementation is provided in the Supplementary Content, Appendix 4 (online).

Finally, a key aspect of any intervention to reduce CAUTI (or other HAIs) is how sustainable the effects are. At the VA Ann Arbor Healthcare System, for example, a team implemented an intervention to reduce use of unnecessary urinary catheters (and thus reduce UTI incidence) in 2010.^135^ This intervention significantly reduced catheter use on medical-surgical wards by 4.6% (absolute difference).^135^ Fowler et al^136^ followed-up 8 years after this intervention, finding that the catheter prevalence had decreased from 13% to 25% (depending on unit type) to 7% (all units). Logistic regression of the data over the entire period (September 2010 to September 2019) indicated that catheter use decreased following intervention (odds ratio, 0.91; 95% CI, 0.86–0.97; *P* = .003). Inappropriate uses of catheters remained low during follow-up and did not appear to be affected by the intervention.^136^ Similarly, Reynolds et al^137^ described a multifaceted CAUTI prevention initiative led by physician and nurse champions in ICUs at Duke University Hospital. The investigators observed a sustained reduction in rates of urine-culture utilization, catheter utilization, and CAUTI over 4 years. Ideally, more studies will assess long-term sustainability when implementing CAUTI prevention initiatives.

The 4-E’s model initially developed by Pronovost et al^118^ to reduce central-line–associated bloodstream infection can also be useful with efforts to reduce CAUTI. Here, using the 4 E’s model, we have outlined the steps that hospitals can use to implement CAUTI reduction programs.

### Engage

Quality improvement projects directed toward improving compliance with CAUTI guidelines have used various techniques to engage the hospital staff to raise awareness of the issue and to increase buy-in.

1. Develop a multidisciplinary team:
  - Physician led^138^
  - Nurse led^138–140^
  - Leadership not specified.^140–146^
2. Involve local champions to promote the program.^137,139,145,147,148^
3. Utilize peer networking.^66,144,148,149^
4. Involve patient and family.

### Educate

Education of the hospital staff can include in-person sessions or educational material available in paper format or electronically. The educational sessions may outline the evidence behind guidelines and the goals of the program and may target specific aspects of CAUTI prevention.

1. Provide education on the following topics:
  - Appropriate catheter care^139–141,145,149–153^
  - Appropriate indications for catheter insertion^138,139,143,147,151,154,155^
  - Insertion technique^141,144,149,151–154^
  - Urine-culture indications, guidance on collection, storage, and transport of urine cultures
  - Hand hygiene education^149^,^151^,^152^
  - Alternatives for indwelling catheters, including to patient and family^66,145,156,157^
  - Management of urinary retention
  - Patient transportation.^66^
2. Provide educational materials as follows:
  - Daily assessment of need for catheter^147,158,159^
  - Decision-making algorithm for catheter indication and urine-culture ordering^139^
  - Case-based education by the infection prevention or stewardship team^139,160^
  - Unit-based educational materials^161^
  - Online learning materials^61,145^
  - Novel cognitive aids—screensaver, hospital leadership memorandum^29^
  - Patient and family educational materials^145^
  - Checklists for resident physicians^162^
  - Simulation training on catheter insertion and maintenance.^61^

### Execute

The process for making quality improvement changes employs new protocols and algorithms. Interventions may be grouped into “bundles” or “checklists” of practices to be implemented simultaneously. The electronic medical record can be leveraged to prompt change in practices. Given the emphasis on quality improvement in physician training programs, engaging resident physicians and other learners in CAUTI prevention efforts may be helpful at teaching hospitals.

1. Standardize care processes as follows:
  - Perform daily assessments of the continued need for urinary catheterization.^157^
  - Provide daily nursing or EMR reminders to physicians to remove unnecessary catheters, often via bedside rounds.^61,138,143,145–147,163–165^
  - Standardize indications for urinary catheter placement.^138,139,141,142,147,157,163^
  - Utilize bladder bundle.^141,148,152,161,166,167^
  - Develop a nurse-driven protocol to discontinue catheter if no longer meeting criteria.^96,140–142,156,168^
  - Optimize the EMR with best-practice order sets, algorithmic decision making for catheter placement, urine cultures.^169^
2. Create reminders that the catheter is in place:
  - Alerts in EMR that a catheter has been placed (Banner, progress note templates)^156,169^
  - Alerts in EMR to remove catheters^156,169^
  - Use cognitive aids that specify line day.^170^
3. Appoint a resident quality champion (in teaching hospitals).^171^
4. Use prewritten or computerized stop orders.^144,171,172^
5. Utilize bladder scanners to measure urine volume prior to inserting a catheter.^139,144,145,156^
6. Standardize products and processes (catheter kit, alternatives to catheters, etc, or catheter maintenance processes).^140,141,144–146^
7. Increase availability of bedside commodes.^145^
8. Conduct individual case reviews (or root-cause analysis) with interprofessional team to identify system issues and practice gaps.^141^
9. Create redundancy of educational materials using the following tools:
  - Posters, screensavers in units^139,144^
  - Pocket cards, apps.^144^

### Evaluate

The success of a CAUTI quality improvement program can be measured by process, outcome, and balancing measures. Most programs have found that providing feedback to the hospital or unit increases awareness.

1. Measure performance or process:
  - Compliance with bundle^149,151,167^
  - Compliance with hand hygiene^149,151,161,167^
  - Urine culture utilization (overall rates of urine culture orders)
  - Catheter utilization (eg: catheter days, SUR, DUR).^108^
2. Provide real-time and routine feedback to staff and leadership:
  - CAUTI rates by ward^140,173^
  - CAUTI rate by hospital^149,151,163^
  - Hand hygiene rate^149,151^
  - Catheter utilization (eg, catheter days, SUR, DUR)^108^
  - Catheter care compliance^149,151^
  - Costs of UTI.^163,174–178^

## Supplementary Material

Supplementary Material (Appendices 1-4)

## Figures and Tables

**Table 1.** Summary of Recommendations to Prevent CAUTI

**Essential practices**
**Infrastructure and resources**
1. Perform a CAUTI risk assessment and implement an organization-wide program to identify and remove catheters that are no longer necessary using 1 or more methods documented to be effective.^34,35,51,52^ (Quality of evidence: MODERATE)
a. Develop and implement institutional policy requiring periodic, usually daily, review of the necessity of continued catheterization.
b. Consider utilizing electronic or other types of reminders (see Supplementary Content, Appendices 2 and 3 online) of the presence of a catheter and required criteria for continued use.^63^
c. Conduct daily review during rounds of all patients with urinary catheters by nursing and physician staff to ascertain necessity of continuing catheter use.^64^
2. Provide appropriate infrastructure for preventing CAUTI.^56^ (Quality of evidence: LOW)
a. Ensure that the supplies for following best practices for managing urinary issues are readily available to staff in each unit, including bladder scanners, non-catheter incontinence management supplies (urinals, garments, bed pads, skin products), male and female external urinary catheters, straight urinary catheters, and indwelling catheters including the option of catheters with coude tips.
b. Ensure that non-catheter urinary management supplies are as easy to obtain for bedside use as indwelling urinary catheters.
c. Ensure the physical capability for urinary catheters with tubes attached to patients (eg, indwelling urinary catheters, some external urinary catheters[EUCs]) to be positioned on beds, wheelchairs, at an appropriate height and without kinking for patients in their rooms and during transport.
3. Provide and implement evidence-based protocols to address multiple steps of the urinary catheter life cycle (Fig. 1): catheter appropriateness (step 0), insertion technique (step 1), maintenance care (step 2), and prompt removal (step 3) when no longer appropriate. (Quality of evidence: LOW)
a. Adapt and implement evidence-based criteria for acceptable indications for indwelling urethral catheter use, which may be embedded as standardized clinical-decision support tools within electronic medical record (EMR) ordering systems. Expert-consensus-derived indications for indwelling catheter use have been developed, although there is limited research that assesses the appropriateness of these uses.^34,65^
4. Ensure that only trained HCP insert urinary catheters and that competency is assessed regularly.^65^ (Quality of evidence: LOW)
a. Require supervision by experienced HCP when trainees insert and remove catheters to reduce the risk of infectious and traumatic complications related to urinary catheter placement.^69–71^
5. Ensure that supplies necessary for aseptic technique for catheter insertion are available and conveniently located. (Quality of evidence: LOW)
6. Implement a system for documenting the following in the patient record: physician order for catheter placement, indications for catheter insertion, date and time of catheter insertion, name of individual who inserted catheter, nursing documentation of placement, daily presence of a catheter and maintenance care tasks, and date and time of catheter removal. Record criteria for removal and justification for continued use. (Quality of evidence: LOW)
a. Record in a standard format for data collection and quality improvement purposes and keep accessible documentation of catheter placement (including indication) and removal.
b. If available, utilize electronic documentation that is searchable.
c. Consider nurse-driven urinary catheter removal protocols for first trial of void without an indwelling catheter when the indication for placement has resolved (see Essential Practices, 3).
7. Ensure that sufficiently trained HCP and technology resources are available to support surveillance for catheter use and outcomes.^73^ (Quality of evidence: LOW)
8. Perform surveillance for CAUTI if indicated based on facility risk assessment or regulatory requirements. as described in Section 5.^73^ (Quality of evidence: LOW)
9. Standardize urine culturing by adapting an institutional protocol for appropriate indications for urine cultures in patients with and without indwelling catheters.^27,53,74–76^ Consider incorporating these indications into the EMR, and review indications for ordering urine cultures in CAUTI risk assessment.^77^ (Quality of evidence: LOW)
**Education and training**
1. Educate HCP involved in the insertion, care, and maintenance of urinary catheters about CAUTI prevention, including alternatives to indwelling catheters, and procedures for catheter insertion, management, and removal.^78^ (Quality of evidence: LOW)
2. Assess healthcare professional competency in catheter use, catheter care, and maintenance.^79–81^ (Quality of evidence: LOW)
3. Educate HCP about the importance of urine-culture stewardship and provide indications for urine cultures. (Quality of evidence: LOW)
a. Consider requiring clinicians to identify an appropriate indication for urine culturing when placing an order for a urine culture.
4. Provide training on appropriate collection of urine. Specimens should be collected and arrive at the microbiology lab as soon as possible, preferably within an hour. If delay in transport to the laboratory is expected, samples should be refrigerated (no more than 24 hours) or collected in preservative urine transport tubes. (Quality of evidence: LOW)
5. Train clinicians to consider other methods for bladder management such as intermittent catheterization, or external male or female collection devices, when appropriate before placing an indwelling urethral catheter. (Quality of evidence: LOW)
6. Share data in a timely fashion and report results to appropriate stakeholders. (Quality of evidence: LOW)
**Infrastructure and resources**
1. Insert urinary catheters only when necessary for patient care and leave in place only as long as indications remain. (Quality of evidence: MODERATE)
2. Consider other methods for bladder management such as intermittent catheterization, or external male or female collection devices, when appropriate. (Quality of evidence: LOW)
3. Use appropriate technique for catheter insertion. (Quality of evidence: MODERATE)
4. Consider working in pairs to help perform patient positioning and monitor for potential contamination during placement. (Quality of evidence: LOW)^88–90^
5. Practice hand hygiene (based on CDC or WHO guidelines) immediately before insertion of the catheter and before and after any manipulation of the catheter site or apparatus. (Quality of evidence: LOW)
6. Insert catheters following aseptic technique and using sterile equipment. (Quality of evidence: LOW)
7. Use sterile gloves, drape, and sponges, a sterile antiseptic solution for cleaning the urethral meatus, and a sterile single-use packet of lubricant jelly for insertion. (Quality of evidence: LOW)
8. Use a catheter with the smallest feasible diameter consistent with proper drainage to minimize urethral trauma but consider other catheter types and sizes when warranted for patients with anticipated difficult catheterization to reduce the likelihood that a patient will experience multiple, sometimes traumatic, catheterization attempts. (Quality of evidence: LOW)
**Management of indwelling catheters**
1. Properly secure indwelling catheters after insertion to prevent movement and urethral traction.^91,92^ (Quality of evidence: LOW)
2. Maintain a sterile, continuously closed drainage system.^92,93^ (Quality of evidence: LOW)
3. Replace the catheter and the collecting system using aseptic technique when breaks in aseptic technique, disconnection, or leakage occur. (Quality of evidence: LOW)
4. For examination of fresh urine, collect a small sample by aspirating urine from the needleless sampling port with a sterile syringe/cannula adaptor after cleansing the port with disinfectant. (Quality of evidence: LOW)
5. Facilitate timely transport of urine samples to laboratory. If timely transport is not feasible, consider refrigerating urine samples or using sample collection cups with preservatives. Obtain larger volumes of urine for special analyses (eg, 24-hour urine) aseptically from the drainage bag. (Quality of evidence: LOW)
6. Maintain unobstructed urine flow. (Quality of evidence: LOW)
a. Remind bedside caregivers, patients, and transport personnel to always keep the collecting bag below the level of the bladder.
b. Do not place the bag on floor.
c. Keep the catheter and collecting tube free from kinking, which can impair urinary flow and increase stasis within the bladder, increasing infection risk.
d. Empty the collecting bag regularly using a separate collecting container for each patient. Avoid touching the draining spigot to the collecting container.
7. Employ routine hygiene. Cleaning the meatal area with antiseptic solutions is an unresolved issue, though emerging literature supports chlorhexidine use prior to catheter insertion.^94–97^ Alcohol-based products should be avoided given concerns about the alcohol causing drying of the mucosal tissues. (Quality of evidence: LOW)
**Additional approaches**
1. Develop a protocol for standardizing diagnosis and management of postoperative urinary retention, including nurse-directed use of intermittent catheterization and use of bladder scanners^98,99^ when appropriate as alternatives to indwelling urethral catheterization. (Quality of evidence: MODERATE)
a. If bladder scanners are used, clearly state indications, train nursing staff in their use, and disinfect between patients according to the manufacturer's instructions.
2. Establish a system for analyzing and reporting data on catheter use and adverse events from catheter use. (Quality of evidence: LOW)
a. Use cumulative attributable difference to identify high-risk units or hospitals as described in Section 5.
b. Measure process and outcomes measures (eg, standardized utilization ratio and standardized infection ratio) as described in Section 5.
c. Define and monitor catheter harm in addition to CAUTI, including catheter obstruction, unintended removal, catheter trauma, or reinsertion within 24 hours of removal.^100^
3. Establish a system for defining, analyzing, and reporting data on non-catheter-associated UTIs, particularly UTIs associated with the use of devices being used as alternatives to indwelling urethral catheters. (Quality of evidence: LOW)
a. Non-catheter-associated UTIs are defined as UTIs that occur in hospitalized patients without an indwelling urethral catheter. These include but are not limited to patients that have had no urinary device at all, as well as those with EUCs, urinary stents, or urostomies, or who undergo intermittent catheterization, that are not captured by the NHSN CAUTI definition.
b. As the incidence of CAUTI continues to decline, the proportion of non-catheter-associated UTIs is increasing in some hospitals.^4^ However, the national incidence of non-catheter-associated UTIs is not known, as surveillance and reporting of these UTIs are not required by US federal agencies.
c. As non-catheter-associated UTIs are a common indication for antibiotics in hospitalized patients, this metric could provide important information as healthcare facilities consider the risks and benefits of newer alternatives to urinary catheters with currently limited published data on adverse events (eg, EUCs for women) to help inform when the benefit outweighs the potential risk for specific patient populations.
**Approaches that should not be used**
1. Routine use of antimicrobial/antiseptic impregnated catheters. (Quality of evidence: HIGH)
2. Breaking a closed system. (Quality of evidence: LOW)
3. Screening for asymptomatic bacteriuria in catheterized patients except in the few patient populations for which this is anticipated to have more benefit than harm, as detailed in the 2019 IDSA Guideline for Management of Asymptomatic Bacteriuria^102^ and the 2019 US Preventative Services Task Force Recommendation on Asymptomatic Bacteriuria in Adults^102^ (eg, pregnant women, patients undergoing endoscopic urologic procedures associated with mucosal trauma). (Quality of evidence: HIGH)
a. Treatment of asymptomatic bacteriuria is not an effective strategy to prevent CAUTI in other patient groups, as it increases the risk of antibiotic-associated complications more than any potential benefit for the prevention of symptomatic CAUTI. The conditions that predisposed the patient to have bladder colonization (anatomic, immunologic) are not resolved by antibiotics, and so the bacteriuria recurs.
4. Catheter irrigation as a strategy to prevent infection. (Quality of evidence: MODERATE)
a. Do not perform continuous irrigation of the bladder with antimicrobials as a routine infection prevention measure.
b. If continuous irrigation is being used to prevent obstruction, maintain a closed system.
5. Routine use of systemic antimicrobials as prophylaxis. (Quality of evidence: LOW)
6. Routine changing of catheters to avoid infection. (Quality of evidence: LOW)
a. In the case of a patient with a long-term catheter in place (ie, >7 days), catheter replacement can be considered at the time of specimen collection for urine testing to obtain a fresh sample.^103,104^
7. Alcohol-based products on the genital mucosa. (Quality of evidence: LOW)
**Unresolved issues**
1. Use of antiseptic solution versus sterile saline for meatal and perineal cleaning prior to catheter insertion.^94–96,105^
2. Use of urinary antiseptics (eg, methenamine) to prevent UTI.
3. Spatial separation of patients with urinary catheters in place to prevent transmission of pathogens that could colonize urinary drainage systems.
4. Standard of care for routine replacement of urinary catheters in place >30 days for infection prevention.^106^
5. Best practices for optimizing and tailoring implementation of CAUTI prevention and urine-culture stewardship from the adult acute-care setting to the pediatric acute-care setting.

**Table 2.** Quality of Evidence^a^

HIGH	Highly confident that the true effect lies close to that of the estimated size and direction of the effect. Evidence is rated as “high” quality when there are a wide range of studies with no major limitations, there is little variation between studies, and the summary estimate has a narrow confidence interval.
MODERATE	The true effect is likely to be close to the estimated size and direction of the effect, but there is a possibility that it is substantially different. Evidence is rated as “moderate” quality when there are only a few studies and some have limitations but not major flaws, there is some variation between studies, or the confidence interval of the summary estimate is wide.
LOW	The true effect may be substantially different from the estimated size and direction of the effect. Evidence is rated as “low” quality when supporting studies have major flaws, there is important variation between studies, the confidence interval of the summary estimate is very wide, or there are no rigorous studies.

a Based on the CDC Healthcare Infection Control Practices Advisory Committee (HICPAC) “Update to the Centers for Disease Control and Prevention and the Healthcare Infection Control Practices Advisory Committee Recommendations Categorization Scheme for Infection Control and Prevention Guideline Recommendations” (October 2019), the Grades of Recommendation, Assessment, Development, and Evaluation (GRADE)^179^ and the Canadian Task Force on Preventive Health Care.^180^

**Table 3.** Process and Outcome Measures for CAUTI and Other Catheter Harms

**Internal reporting**			
**Process measures: Ongoing measurement of recommended CAUTI prevention practices**			
Conduct random audits of selected units and calculate utilization ratios		(No. of patients on the unit with a urinary catheter and an inappropriate indication for the catheter/no. of patients on the unit with a urinary catheter in place)x100 = % inappropriate catheters based on insertion documentation	
Measure the percentage of patients with an indwelling urethral catheter and documentation of daily assessment of need		(No. of patients with an indwelling urethral catheter who have documentation of daily assessment/no. of patients with an indwelling urethral catheter)x100 = % compliance with daily documentation of continued need for indwelling urethral catheter	
Conduct audits of all units and calculate the device utilization ratio^109^		(Total no. of urinary catheter days for all patients in a unit who have an indwelling urethral catheter/total no. of patient days for all patients in the unit who are monitored) = point prevalence of presence of indwelling urethral catheters for a specific unit	
**Outcome measures**			
Measure CAUTI rates over time to gauge the longitudinal impact of prevention strategies.^34^ Patient days may be better suited as a denominator in locations with significant reductions in catheter use or changes in risk profile.^111^		(No. of CAUTIs in a unit that is monitored/total no. of urinary catheter days for all patients on the unit who have an indwelling urethral catheter)x1,000 = cases per 1,000 catheter days (No. of CAUTIs in a unit that is monitored/total no. of patient days for all patients in a unit that is monitored)x10,000 = cases per 10,000 patient days	
Standardized infection ratio (SIR): adjusted summary measure that allows for comparison to the national benchmark and can be used to track CAUTI incidence over time. Useful for evaluating relative risk differences.		Observed no. of CAUTIs/predicted no. of CAUTIs Predicted no.: the estimated number of CAUTIs based on data reported to NHSN during a baseline period	
Cumulative attributable difference (CAD): to target prevention efforts on hospitals or units with the highest excess CAUTIs to reach the goal SIR. Cost-effective and useful for evaluating absolute risk differences.		Observed no. of CAUTIs - (predicted no. of CAUTIsxSIR_goal_) = CAD	
**External reporting**			
**Outcome measures**			
Standardized utilization ratio (SUR): the primary summary measure used to compare device utilization at the national, state, or facility level.		Observed no. of device days/predicted no. of device days	
Population standardized infection ratio (pSIR): accounts for both SIR and SUR, reflecting device care and device utilization		Observed no. of CAUTIs for a population ^ predicted no. of CAUTIs the population	
• Facilities with low SIR and high SUR may represent low-risk catheter use, better maintenance and care of indwelling catheters, or strict urine-culturing practices. These facilities may benefit from focusing prevention efforts on decreasing device utilization to account for noninfectious harms as well. • Facilities with high SIRs and low SUR may represent a population with catheter use in higher-risk patients, inadequate catheter care, or indiscriminate urine-culturing practices. These facilities may benefit from focusing on catheter maintenance and culturing stewardship.^107,108^			
Other catheter harms			
Measure	Type of Measure	Type of Reporting	Outcome Impacted
Compliance with insertion indication documentation	Process	Internal	CAUTI Catheter harm
Compliance with documentation for daily need of catheter	Process	Internal	CAUTI Catheter harm
Device utilization ratio (DUR)	Process	Internal	CAUTI Catheter harm
CAUTI rate (denominator = catheter days or patient days)	Outcome	Internal	CAUTI
Cumulative attributable difference (CAD)	Outcome	Internal	CAUTI
Standardized utilization ratio (SUR)	Outcome	External	CAUTI Catheter harm
Standardized infection ratio (SIR)	Outcome	Internal and external	CAUTI

**Table 4.** Criteria, Advantages, and Limitations of Definitions Used for Identifying CAUTIs^a^

Definition Type	Criteria	Strengths	Limitations
NHSN CAUTI definition	Significant bacteriuria (≥10^5^ CFU/mL) with ≤2 organisms; in addition to at least 1 of the following clinical findings: fever, suprapubic tenderness, costovertebral angle pain or tenderness; if catheter is removed, urgency, frequency, dysuria can also be used^46,181^	Objective criteria, reproducible, universally used for quality improvement initiatives	Does not correlate with clinical CAUTI or clinician practice; heavily dependent on fever even if alternate source present; limited in evaluating device use and care
IDSA CAUTI definition	Bacteriuria (≥10^3^ CFU/mL); signs or symptoms referable to the genitourinary tract, and no other identified source of infection^35,87^	Clinical; independent of urinalysis results; the diagnosis of exclusion minimizes overdiagnosing CAUTI in patients with asymptomatic bacteriuria	Not easy to use or apply to a specific patient; runs counter to ingrained diagnostic biases (pyuria not used)
Claims-Based CAUTI definition	Administrative claims data for the purposes of identifying UTIs as healthcare- and catheter-associated	Identified by diagnosis codes submitted by hospital coders routinely in the process of generating and submitting administrative data to request hospital payment	Low sensitivity to capture clinical CAUTIs (ie, many CAUTIs that occur are identified simply as UTIs but not CAUTIs in claims data
Revised McGeer criteria (used in long-term care)	Localizing urinary tract signs with or without fever and leukocytosis in addition to microbiological criteria with bacteriuria ≥10^5^ CFU/ml of ≤2 organisms^182^	The revised criteria include microbiologic criteria and account for the low probability of UTI in residents without indwelling catheters in the absence of localizing symptoms	Microbiological criteria for catheter urine specimens are less specific.

a Adapted from Advani SD, Fakih MG. Health Care-Associated Urinary Tract Infections (Including CAUTI), Mayhall’s Hospital Epidemiology and Infection Prevention^45^

**Table 5.** CAUTI Literature

Systematic review	1. A systematic review in hospitalized patients reported that the use of an intervention including a reminder to staff that a catheter was in place and/or a stop order to prompt removal of unnecessary catheters reduced the CAUTI rate by 53%.^12^ 2. A systematic review reported that evidence did not support routine use of indwelling bladder catheters for caesarean section.^183^ 3. A Cochrane review and meta-analysis of bladder washout policies to prevent blockage of long-term catheters in adults concluded evidence was too scanty to conclude whether there were benefits.^184^ Trials were generally of poor quality or were incompletely reported.
Routine postoperative indwelling urethral catheter	A prospective randomized trial of thoracic surgery patients managed with epidural analgesia compared morning after surgery catheter removal with catheter remaining in place as long as the thoracic epidural analgesia was functioning. There was a longer time to reach postvoid residuals of <200 mL with early removal but no increased need for recatheterization. CAUTI rates were not reported.^185^
Catheter materials	A prospective randomized 3-arm trial in 24 UK National Health Service (NHS) hospitals compared a standard latex catheter, latex silver alloy-coated catheter and a silicone nitrofurazone impregnated catheter.^186^ The rates of symptomatic culture confirmed urinary infection at 6 weeks were similar in patients who received either of the 2 latex catheters; a small decrease in rates was noted for patients with the nitrofurazone silicone catheter (OR, 0.68; 97.5% CI, 0.48–0.99; *P* = .017). It is not clear whether the difference was attributable to the silicone or the antimicrobial agent. The nitrofurazone catheter was associated with greater patient discomfort (OR, 1.39; 97.5% CI, 1.13–1.60) and increased catheter removal (OR, 1.77;97.5% CI, 1.51–22.07). A cost analysis suggested universal use of a nitrofurazone catheter might be cost-effective in the NHS system, but the analysis was compromised by uncertainty in length of stay estimates.^187^
Infrastructure requirements	The prevention programs reported have varied in components and implementation approaches, and usually multiple interventions have been implemented simultaneously. Decreasing catheter use through restricted indications for placement or duration of catheterization are major components for most programs. All studies used a pre-post intervention trial design. 1. A restrictive urinary catheter policy together with daily review of necessity and discussion of appropriateness of new catheter insertions with emergency medicine and internal medicine physicians decreased catheterization from 17.5% to 6.6% of patients.^155^ 2. A statewide program in Michigan focused on educating clinicians about appropriate urinary catheter indications and included daily assessment of continued catheter need during nursing rounds. There was a decrease in catheter use from 18.1% to 13.8%, whereas the proportion of catheters with appropriate indications increased from 44% to 58%.^188^ 3. A survey-based study compared a random sample of US hospitals to all Michigan hospitals and reported that the Michigan hospitals more frequently participated in collaboratives to reduce HAIs, used bladder scanners to estimate bladder volumes, and used catheter reminders or stop orders and/or nurse-initiated discontinuation. More frequent use of these practices coincided with a 25% reduction of CAUTI rates in Michigan, as compared to a 6% reduction in non-Michigan hospitals.^119^ 4. Resident peer-to-peer education for compliance with emergency department urinary catheter placement indications resulted in increased knowledge 3 months following an educational intervention, but there were no differences in catheter use or the proportion of catheters meeting appropriate indications.^189^ 5. An educational intervention incorporating catheter indications, timely removal, and correct management together with initiation of active CAUTI surveillance, resulted in a decrease in catheterization rates from 18.5% to 9.2% (*P* < .05), and a nonsignificant decrease of CAUTI from 6.6 per 1,000 catheter days to 5.8 per 1,000 catheter days.^150^ 6. Introducing a UTI bundle (avoidance of catheter insertion, maintenance of sterility, product standardization, early catheter removal) in a single-center neurologic ICU significantly decreased catheter utilization from 100% to 73% and CAUTI from 13.3 to 4.0 per 1,000 catheter days.^141^ 7. A CAUTI prevention program including education, implementation of common CAUTI prevention practices, outcomes and process measures, and feedback of CAUTI outcomes and process measures was implemented in pediatric ICUs in 6 developing countries, and reported a decrease in CAUTI rates from 5.9 to 2.6 per 1,000 catheter days (RR, 0.43; 95% CI, 0.21–1.0).^149^
Implementing programs to prevent CAUTI	1. A multicenter qualitative study identified 4 recurrent themes relevant to hospital use of prevention practices: recognizing value of early catheter removal; focus on noninfectious complications and presence of a “champion”; hospital specific pilot studies for devices; and external forces such as public reporting.^190^ 2. A statewide initiative in Michigan introduced a bladder bundle to decrease CAUTI using a collaborative model and strategies to facilitate implementation including “engage and educate,” “execute,” and “evaluate.”^191^ 3. A qualitative assessment in 12 hospitals in Michigan of perceptions and key issues influencing implementation of CAUTI prevention practices identified difficulty with nurse and physician engagement, patient and family request for indwelling catheters, and catheter insertion practices and customs in the emergency department as common barriers.^131^ 4. A 2-tiered approach of evidence-based strategies to CAUTI prevention (see the example in Fig. 3 from a recent CDC collaborative) including a large focus on catheter and urine test stewardship implemented primarily as an externally facilitated educational and data feedback intervention has also been recently evaluated in 2 large national collaboratives, which targeted units with higher-than-average CAUTI rates at baseline. Unfortunately, neither collaborative yielded significant reductions in NHSN-reported CAUTI rates or urinary catheter device utilization beyond secular trending reductions.
Surveillance	1. A simulation model comparing denominators of catheter days and patient days reported that CAUTI rates were reduced for 93 of 100 simulations. In 27% of stimulations, the CAUTI rate (with catheter days as denominator) increased while all others showed greater decreases with a denominator of patient days rather than catheter days.^192^ 2. Data extracted from electronic chart review were 100% sensitive and 98% specific compared with bedside review to verify the type and presence of a urinary catheter at 1 VA hospital.^182^
