# Supplementary material for: Strategies to prevent catheter-associated urinary tract infections in acute-care hospitals: 2022 Update
Source: Infect Control Hosp Epidemiol. Author manuscript; Available in PMC 2024 Jul 30. (PMC11287483; doi:10.1017/ice.2023.137)
Supplement: Supplementary Material (Appendices 1-4) [file NIHMS2009542-supplement-Supplementary_Material__Appendices_1-4_.docx]

# Supplementary content

## Appendix 1. Published guidelines and recommendations for prevention of infections associated with short-term indwelling urethral catheters

|  | **Centers for Disease Control and Prevention (2009)^18^** | **IDSA (2009)** | **Epic 2 Project**  **NHS, UK (2007)^20^** |
| --- | --- | --- | --- |
| Documentation of catheter insertion | C | ND | Yes |
| Trained HCP | Yes | ND | Yes |
| Train patients and family | Yes | ND | Yes |
| Hand hygiene | Yes | ND | Yes |
| Evaluation of necessity | Yes | Yes | Yes |
| Evaluation of alternative methods | Yes | Yes | Yes |
| Regular review of ongoing need | Yes | Yes | Yes |
| Choice of catheter | U | U | U |
| Use smallest gauge catheter | Yes | ND | Yes |
| Aseptic technique/sterile equipment | Yes | Yes | Yes |
| Barrier precautions for insertion | Yes | ND | ND |
| Antiseptic cleaning of meatus | U | ND | No |
| Secure catheter | Yes | ND | No |
| Closed drainage system | Yes | Yes | Yes |
| Obtain urine samples aseptically | Yes | ND | Yes |
| Replace system if break in asepsis | Yes | ND | ND |
| No routine change in catheter | Yes | U | Yes |
| Routine hygiene for meatal care | Yes | Yes | Yes |
| Avoid irrigation for purpose of preventing infection | Yes | Yes | Yes |
| Separate patients with catheters | U | U | ND |
| Use of preconnected system | C | C | ND |
| Performance feedback | C | ND | ND |
| Rates of CAUTI and bacteremia | C | C | ND |

Note: C, consider; CAUTI, catheter-associated urinary tract infection; IDSA, Infectious Diseases Society of America; NHS, UK National Health Service; ND, not discussed; U, unresolved (choice left to individual and patient factors)

## Appendix 2. Urinary catheter reminder

“A Reminder Reduces Urinary Catheterization in Hospitalized Patients,” Saint S, Kaufman SR, Thompson M, Rogers MAM, Chenoweth CE, The Joint Commission Journal on Quality and Patient Safety^1^


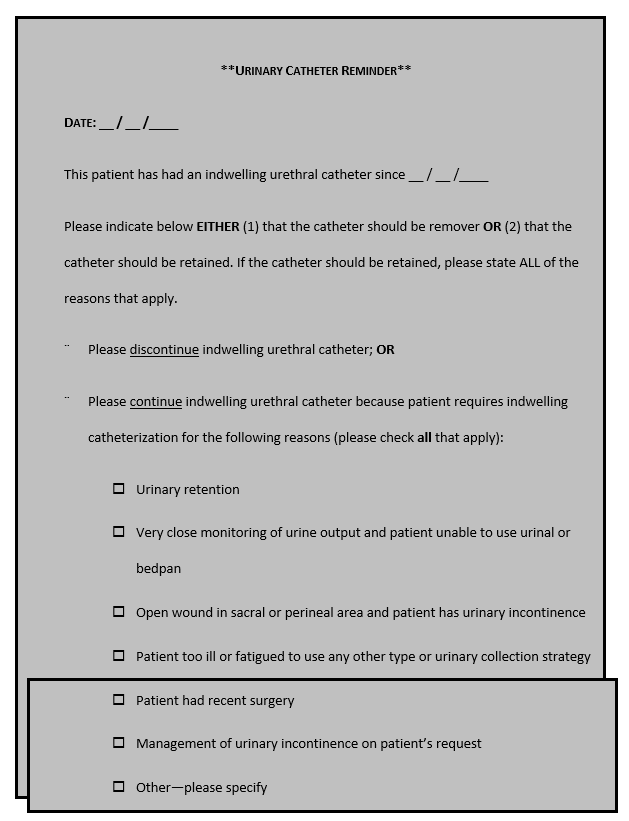


## Appendix 3. Fundamental elements of accountability for HAI prevention

| - Senior management are responsible for ensuring that the healthcare system supports an infection prevention and control (IPC) program that effectively prevents healthcare-associated infections (HAIs) and the transmission of epidemiologically important pathogens |
| --- |
| - Senior management is accountable for ensuring that an adequate number of trained HCP are assigned to the IPC program, and adequate staffing of other departments that play a key role in HAI prevention (e.g., environmental services) |
| - Senior management is accountable for ensuring that HCP, including licensed and non-licensed HCP, are adequately trained and competent to perform their job responsibilities |
| - Direct HCP (e.g., physicians, nurses, aides, and therapists) and ancillary personnel (such as environmental service and equipment processing personnel) are responsible for ensuring that appropriate IPC practices are used at all times (including hand hygiene, standard and isolation precautions, and cleaning and disinfection of equipment and the environment) |
| - Senior and unit leaders are responsible for holding personnel accountable for their actions |
| - IPC leadership is responsible for ensuring that an active program to identify HAIs is implemented, that HAI data are analyzed and regularly provided to those who can use the information to improve the quality of care (e.g., unit staff, clinicians, and hospital administrators), and that evidence-based practices are incorporated into the program |
| - Senior and unit leaders are accountable for ensuring that appropriate training and educational programs to prevent HAIs are developed and provided to HCP, patients, and families |
| - HCP from the IPC program, the laboratory, and information technology departments are responsible for ensuring that systems are in place to support the surveillance program |

## Appendix 4. Problem-solving for implementation of programs to prevent CAUTI

Created by Sarah Krein, Jane Forman, Christine Kowalski, Laura Damsehroder, Molly Harrod, Milisa Manojlovich and Sanjay Saint for [www.catheterout.org](http://www.catheterout.org). Additional resources available at [www.catheterout.org](http://www.catheterout.org).

| **BARRIERS** | **POSSIBLE SOLUTIONS** | |
| --- | --- | --- |
| **ENGAGEMENT** | | |
| **Some may not be on board** with indwelling urethral catheter removal | - Get buy-in before implementation. For example, ask “who do we have to convince on this floor?” Have that person help to develop the plan or participate in the education for that unit. - Listen to nurses’ concerns and address them to nurses’ satisfaction - Provide education and equipment/materials to facilitate alternative nursing strategies for incontinence care | |
| **Lack of/or problems with nurse champions**   - Nurse managers tell your team that they are “too busy” to implement the new practice. - Individuals identified as champions do not go out on the unit and do not have direct contact with inpatients. | - Identify the types of champions that work in your organization. Not a one-size-fits-all strategy. For example:   - Use nurse educators as champions   - Have more than one nurse champion, e.g., co-champions, all nurse managers and educators.   - An LPN can be the champion if s/he is someone who others on the unit respect and go to for advice. - Recognize nurse champions via such mechanisms as certificates of recognition, annual evaluation appraisals, newsletters, notifying CNO. | |
| **Lack of physician buy-in to new practice or physicians are resistant to change in general**   - Do not see indwelling urethral catheters as a risk. - “Way down on their priority list.” - Can’t get physicians to buy in to the new practice bundle because they do not want “to make waves”. | - Provide data about urinary catheter use, feedback to physicians about monthly indwelling urethral catheter prevalence & CAUTI rates. - Provide one-on-one education (evidence-based and patient safety oriented). - Engage medical leadership support, e.g., chief of staff. - Involve physicians as much as possible in planning, education, and implementation; include physicians on your team. - Identify a physician champion who will:   - Meet with other physicians to get them on board.   - Back up nurses when there’s a disagreement.   - Conduct CME. Present evidence, e.g., highlight how often physicians have a patient with an indwelling urethral catheter and forget. | |
| **Lack of physician champion** | - In some institutions, physicians may tend to go along with nurse recommendations so they rely heavily on nurse champions. The new practice could be seen as a “nursing initiative.” - Also see Lack of physician buy-in above. | |
| **Leadership does not see CAUTI as a priority** | - Prepare and present a business case to help convince leadership the time and cost factors for implementing the new practice would be worth it. Present a good business case. - Remind leadership about CMS non-payment rule. - Be sure leadership gets monthly CAUTI/catheter use data. | |
| **Large size hospital makes unit-to-unit roll-out difficult** | - Create unit-based teams with stakeholders from different units/depts. | |
| **General guidance** | - Get people on the team who feel CAUTI is worth working on. - Highlight staff who have adopted the new practice. - Know the system and how to get practice changes through relevant committees. | |
| **EDUCATION** | |  |
| **Gaps in knowledge of infectious and non-infectious consequences of CAUTI for patients**   - CAUTI not seen to be as serious as other infections - Belief that since the patient is going to be on bed rest the catheter is indicated. - Not thinking about an indwelling urethral catheter as an invasive device or as a less risky device compared to other devices, such as central venous catheters. - Belief that catheters are helpful to prevent development of pressure ulcers. | - Content   - Distribute Signs and pocket guides with insertion/DC criteria   - Share safety and quality literature - Options on how to educate staff   - Create tailored educational materials. Different materials for IPs, nurses, physicians, clinical leadership, and perhaps for each unit, depending on what motivates staff in that unit (e.g., decrease length of stay, ambulate patient, decrease UTI risk).   - Nurses:     - Education mandated by nurses’ direct supervisor     - Educate on the floor, in grand rounds, other venues.   - If it’s difficult to educate all staff, as in a large hospital, create computerized education modules |  |
| **Not knowing what to do to prevent CAUTI** | - HICPAC guidelines |  |
| **Nurses’ time is often overcommitted, so difficult to do education**   - Overtime not allowed. - No “dedicated” time away from patient care. | - Rather than having the nurses attend education sessions, bring the education to the bedside. E.g., doing competencies on the unit; talking with nurses one-to-one during the point prevalence assessments. - Incorporate education on CAUTI into annual competency testing (e.g., at the same time that CPR is renewed). |  |
| **EXECUTE** | |  |
| **ELIMINATE UNNECESSARY PLACEMENT OF INDWELLING URETHRAL CATHETERS** | |  |
| **Non-indicated indwelling urethral catheters inserted in ED**   - Indwelling urethral catheter is inserted with no order written. When patient gets to the floor, nurses and physicians are unaware of catheter’s presence or reason it was placed. - ED nurses think they are doing the floor nurses a favor by inserting the indwelling urethral catheter and assume that the patient might need it. - ED nurses using catheter for specimen collection and then leaving it in place. - Alternate practices (e.g., closed straight catheter system) eliminated due to cost. | - Involve ED medical and nursing directors as champions or supporters of practice change. - Work with ED to put a process in place that assures that an order was written and appropriate indications for use are followed. - Education about indications for insertion for the ED nurses and physicians. - Re-implement alternative practice (e.g., closed straight catheter system). - Create notification system to inpatient admitting team that a catheter was placed in the ED to prompt discontinuation order or documentation of an appropriate indication to keep catheter. |  |
| **No catheter policy regarding insertion standards in place** | - Develop a policy on catheter insertion indications |  |
| **Patient request**  Clinicians give in to patient or family requests for indwelling urethral catheter, or believe that the patient wants the catheter in. | - Discuss risks of indwelling urethral catheters with patients and families. - Review documentation of the rationale for placement if indications are not met and reinforce use of appropriate indications. |  |
| **Lack of physician buy-in once the new practice is initiated.**  See Lack of Physician Buy-in, above | - Collect and report data on catheter use and CA-UTI physician/service: physicians may be responsible to type of data collection - Meet with resisting physician to address concerns. - Consider involving resisting physicians in potential champion role, as accountable for catheters and CA-UTI rates. |  |
| **ENSURE PROPER INSERTION TECHNIQUE** | |  |
| Non-Aseptic Insertion Technique   - By nurses, aides, nursing care assistants, medical students. | - Develop competencies for those who insert catheters. - Restrict catheter insertion practice to RNs. - Develop a policy on catheter insertion techniques if none is in place. |  |
| **TIMELY DISCONTINUATION OF INDWELLING URETHRAL CATHETERS** | |  |
| **Nursing workload**   - Nurses are concerned that they will have to spend more time cleaning up patients if the indwelling urethral catheter is removed - General feeling of being overworked (“just trying to get through my shift”) - What you might see:   - Nurses tell the physician or other nurses, “I do not want this catheter out” or that the physician needs I’s and O’s).   - Especially problematic on weekends – no one is monitoring catheter removal. | - Monitor   - Catheter patrol: daytime charge nurses monitor which patients have indwelling urethral catheters, assisting with toileting, and assess indications. If not indicated, talk with bedside nurse or ask physicians to DC.   - Daily assessment tool: bedside nurse assesses indications for continued use and if not indicated, nurses discuss removal with physician. - Feedback:   - Data board in nurse units w/monthly indwelling urethral catheter prevalence and CAUTI rates. - Nurse aides delegated to prioritize toileting activities over other activities (e.g., stocking supplies or cleaning equipment). - Share experiences where nurses report positive experiences from catheter removal programs. |  |
| **Shift schedules hamper communication among nurses**  3-day, 12-hour shifts and block schedules can make it difficult to share information across shifts and departments. | - Identify a nurse champion on each shift. |  |
| **No catheter policy on discontinuation in place** | - Develop a policy on discontinuation |  |
| **Patient or family request**   - Nurses and/or physicians believe their patients want to keep the catheter. Some patients do (e.g., because they are incontinent or don’t want to get out of bed), and will ask their nurses and physicians to keep it in even if it’s not indicated | - Discuss risks of indwelling urethral catheters with patients and families - Review documentation of the rationale for use and reinforce use of appropriate indications. |  |
| **Patient Safety: Balancing risk of falls**.   - Competing priorities: Well-intended misconception that urinary catheters prevent falls, as a fall is a “never event” is also being assessed as a quality measure. | - Institute fall prevention strategies, for example:   - Instruct the patient to request assistance.   - Provide patient with non-skid footwear.   - Ensure that path to restroom is free of obstacles   - Evaluate chair and bed height.   - Ensure that assistive devices (if being used) are within patient reach   - Engage patient and family in efforts to provide assistance as needed.   - Other strategies as determined by nursing care plan and institutional policy. - Incorporate urinary management (e.g., planned toileting) as part of broader fall prevention program. - Remind nurses that urinary catheters do not reduce fall rates, and can increase fall rates by tripping over catheters. |  |
| **Nurses are not confident speaking with physicians about removal.** | - Find a physician champion to support nurse requests for removal. - Nurse manager prompts nurses to speak with physicians. - Education on communication. - Identify if nurses are reluctant to speak to all physicians, or just particular physicians: tailor solution if individual physicians resist catheter removal. - If nurses are confident to place catheters autonomously (without borders) but not removal, highlight this contradiction. |  |
| **Physician resistance to nurses discontinuing indwelling urethral catheters using an automatic stop order.** | - Nurses prompt physicians for DC order as an initial strategy to build rapport. - Identify a physician champion who can act as an advocate. |  |
| **Lack of physician buy-in** | See Lack of MD buy-in, above |  |
| **Resistance to early indwelling urethral catheter removal – Surgeons and Urologists** | - Physician champion presents at med staff meeting about indwelling urethral catheter indications and non-indications. - Work with the physician assistants to DC indwelling urethral catheters on day 2 after surgery. - Engage a surgeon and/or urologist as a physician champion and work with then to establish conditions under which the catheter can be retained. - Recognize that urology patients may have unique and appropriate indications for placing and keeping catheters beyond the HICPAC indications.   Also see Lack of Physician Buy-in, above |  |
| **Indwelling urethral catheters left in when patient is transferred within the hospital** (e.g., catheter placed in surgery, patient goes up to ICU, then to floor) | - Establish process to ensure that all lines and devices are reviewed and removed (if appropriate) prior to transfer. - Consider changes to transfer forms to include information about catheter presence, date of insertion, indication. |  |

# References

1. Saint S, Kaufman SR, Thompson M, Rogers MA, Chenoweth CE. A reminder reduces urinary catheterization in hospitalized patients. Joint Commission journal on quality and patient safety 2005;31(8):455-62. DOI: 10.1016/s1553-7250(05)31059-2.
